# POCUS for Visualization and Facilitation of Urinary Catheter Placement

**DOI:** 10.24908/pocus.v5i2.14431

**Published:** 2020-11-18

**Authors:** Joseph Garagliano, Jai Madhok

**Affiliations:** 1 Department of Anesthesiology, Perioperative, and Pain Medicine, Stanford University School of Medicine Stanford, California

**Keywords:** Urinary catheter, Foley, BPH

## Introduction

The use of point-of-care genitourinary ultrasound allows dynamic visualization of urinary catheter placement within the bladder and serves to minimize the potential for traumatic injury to the prostate and urethra during difficult insertion. 

## Case Presentation

A 61-year old man with pertinent past medical history of benign prostatic hyperplasia (BPH) was admitted for bilateral L1-L3 laminoforaminotomy for symptomatic lumbar stenosis. Routine intraoperative urinary catheter placement with a 14 French Coudé catheter was attempted by operating room (OR) nursing staff following induction and intubation. The catheter was placed, and the balloon was inflated without resistance. However, there was no return of urine immediately or in the hour following placement even though the patient had reported the urge to urinate prior to induction of anesthesia. A point-of-care ultrasound of the bladder and prostate was performed demonstrating approximately 200 mL of urine retained in the bladder. 

There was high suspicion for improperly placed catheter even though the initial placement was uncomplicated, and the catheter was inserted up to its hub. Ultrasound of the bladder and prostate failed to show the catheter balloon in the bladder. Dynamic manipulation of the catheter under POCUS showed its tip repeatedly abutting and traumatizing the prostate at the bladder neck without advancement into the bladder (see online Video S1). 

At this point urology was consulted for difficult urinary catheter placement. Repeat placement of both 14 and 18 French Coudé catheters was attempted without return of urine and complicated by trauma of the genitourinary tract evidence by blood at the meatus. Flexible cystoscopy was performed to assist visualization of the urethra but was complicated by blood and clots obscuring the view. Decision was made to switch to a flexible ureteroscope to gain access into the bladder. Given the degree of trauma, anatomic challenges, and bleeding in the prostatic urethra, visualization via the flexible ureteroscope was limited so real-time POCUS was used to slowly advance the flexible ureteroscope into the bladder while minimizing trauma to the prostate urethra, A guidewire was then advanced through the ureteroscope under direct visualization into the bladder (see online Video S2). The ureteroscope was removed and a 16 French council tip catheter was advanced over the guidewire into the bladder with efflux of clear urine. The balloon was inflated with proper catheter tip placement in the bladder confirmed by ultrasound (see online Video S3). 

## Bladder Ultrasonography

The bladder is typically imaged using a 3.5 – 5MHz transducer via a transabdominal suprapubic approach. The bladder is best visualized when full. On transverse imaging, the normal urinary bladder appears as an almost rectangular shape with thin walls (< 4mm in thickness). Sagittal images of the bladder can be obtained by rotation the probe 90 degrees from the transverse plane and a normal bladder usually appears triangular in shape. The bladder outlet can be visualized by tilting the tail of the probe down towards the umbilicus when in the transverse position. A normal urine-filled bladder with no clots or masses appears completely anechoic within the walls. Bladder volume is estimated using the formula 1

V = 0.7 (w * d * h)

where w = maximum diameter in transverse plane, d = maximal diameter in sagittal plane, and h = maximum depth in sagittal plane.

## Discussion

This case illustrates the importance of genitourinary POCUS in visualizing proper or improper placement of a urinary catheter, estimating bladder volume, and avoiding prostatic and urethral trauma during difficult catheter placement. Even routine urinary catheter placement subjects the patient to risks of trauma and infection 2. This risk is increased in patients with BPH as the prostate compresses the prostatic urethra and makes passage of a urinary catheter more difficult 3. In this case of a patient with known BPH, passage of an intraoperative urinary catheter appeared to be uncomplicated, yet there was no efflux of urine even with the bladder holding 200 mL urine. 

Failure of a urinary catheter to drain urine after uncomplicated placement can be caused by an empty bladder, placement of catheter tip in the urethra, clogged catheter tip, kinked catheter, or creation of a false passage. The most severe complications is creation of a false passage, as this can result in pain, abscess formation, urethrocutaneous fistula, and infection 4. The use of POCUS to facilitate placement of a urinary catheter over a hydrophilic guidewire in a patient with BPH and prior placement of suprapubic catheter has been reported 5. Further, POCUS has been used to dynamically guide a Foley catheter into the uterine cavity to tamponade life-threatening post-procedure hemorrhage 6.

This case demonstrates the role of POCUS in visualizing the tip of a urinary catheter when the catheter fails to efflux urine after placement and can differentiate from false passage versus kinked catheter versus inadequate tip advancement into the bladder. Repeated attempts at re-insertion in the absence of direct ultrasound visualization can result in trauma, bleeding, and edema requiring indwelling Foley catheter for a prolonged period of time. Furthermore, flexible video cystoscopy may not be available and bladder POCUS offers an alternative visualization method. Lastly, if trauma is suspected, cystoscopy may be of limited utility as blood and clots may impair the cystoscope’s view. POCUS offers visualization of urinary catheter within the bladder and prostate even in the setting of a urethral trauma. 

## Conflicts of Interest

None declared.

## Supplementary Material

Video S1 POCUS of urinary catheter tip failing to advance into bladder with traumatization of prostate and urethra.

Video S2 POCUS of flexible ureteroscope successfully passing into bladder.

Video S3 POCUS of urinary catheter advanced over guidewire successfully passing into bladder.
